# Clinical phenotyping is key to differentiating *RFC1*-associated neuropathy from immune-mediated neuropathy

**DOI:** 10.1093/braincomms/fcae212

**Published:** 2024-06-15

**Authors:** Sue-Faye Siow, Kishore Raj Kumar

**Affiliations:** Department of Clinical Genetics, Royal North Shore Hospital, St Leonards 2065, Australia; Sydney Medical School, University of Sydney, Camperdown 2050, Australia; Sydney Medical School, University of Sydney, Camperdown 2050, Australia; Translational Neurogenomics Group, Genomic and Inherited Disease Program, Garvan Institute of Medical Research, Darlinghurst 2010, Australia; Molecular Medicine Laboratory and Department of Neurology, Concord Repatriation General Hospital, Concord 2139, Australia; School of Clinical Medicine, UNSW Medicine & Health, University of New South Wales, Kensington 2052, Australia

## Abstract

This scientific commentary refers to ‘Pathologic *RFC1* repeat expansions do not contribute to the development of inflammatory neuropathies’, by Nagy *et al*. (https://doi.org/10.1093/braincomms/fcae163).

**This scientific commentary refers to ‘Pathologic *RFC1* repeat expansions do not contribute to the development of inflammatory neuropathies’, by Nagy *et al*. (https://doi.org/10.1093/braincomms/fcae163).**

Biallelic pathogenic intronic AAGGG repeat expansions in the *RFC1* gene were found to be associated with cerebellar ataxia, neuropathy, vestibular areflexia syndrome (CANVAS) in 2019.^1^ Since then, several groups have identified biallelic *RFC1* repeat expansions in a small number of patients previously diagnosed with immune-mediated neuropathies.^2‐4^ These findings raise the question of whether patients with inflammatory neuropathies should be screened for *RFC1* repeat expansions. The study by Nagy *et al*.^5^ in this issue of *Brain Communications* sought to answer this question by screening a large number of patients (*n* = 259) with various forms of immune-mediated neuropathies for pathogenic *RFC1* repeat expansions.

Unlike the preceding groups, Nagy *et al.*^5^ did not identify any patients with biallelic pathogenic *RFC1* repeat expansions within their cohort. In addition, they found that the carrier frequency of pathogenic *RFC1* repeat expansions in their cohort was comparable to the general population. Their findings may be attributed to differences in clinical presentation of *RFC1*-associated neuropathy compared to immune-mediated neuropathy (Table 1). Typically, *RFC1*-associated neuropathy is a slowly progressive, sensory predominant axonal neuropathy with little to no motor involvement.^1,2^ In contrast, immune-mediated neuropathies usually present with mixed sensorimotor involvement, with chronic inflammatory demyelinating polyneuropathy (CIDP), the most common chronic immune-mediated neuropathy, presenting with a demyelinating picture.^6^ Nagy *et al.*^5^’s cohort consisted of patients with CIDP, multifocal motor neuropathy (MMN) and acute inflammatory demyelinating neuropathy (AIDP), all conditions with a different clinical phenotype to that of *RFC1*-associated sensory neuropathy. In Hirano *et al.*^4^’s cohort, patients who were found to have biallelic pathogenic *RFC1* expansions (4 out of 240) were diagnosed with Guillain–Barre syndrome (GBS), idiopathic sensory ataxic neuropathy, myelin-associated glycoprotein neuropathy and sensory autonomic neuropathy, all conditions with a predominantly sensory presentation though motor involvement was also present in 3 out of 4 patients. Similarly, 7 out of 42 patients with chronic idiopathic axonal neuropathy from Currò *et al*.^2^’s cohort with biallelic *RFC1* pathogenic variants were previously diagnosed with an inflammatory cause including CIDP, Sjogren’s syndrome, vasculitis, post-infectious and paraneoplastic. Therefore, clinical presentation, nerve conduction study findings and nerve biopsy results are likely able to differentiate patients who have an immune-mediated neuropathy from those who have a sensory axonal neuropathy that could be associated with *RFC1* (Table 1, note that no new data was generated in support of this commentary).

**Table 1:** *RFC1*-associated neuropathy versus immune-mediated neuropathy^1,2,3,7^

	*RFC1*-associated neuropathy	Immune-mediated neuropathy (includes CIDP, GBS and vasculitic neuropathy)
Clinical presentation	Slowly progressive sensory neuropathy with little to no motor involvement. Can be associated with cerebellar signs, bilateral vestibular areflexia, dysautonomia and chronic cough.	Acute or chronic depending on subtype with mixed sensorimotor involvement.
Nerve conduction study findings	Non-length-dependent axonal sensory neuropathy: absent or reduced sensory action potentials, normal or slightly decreased sensory conduction velocities. Normal motor conduction.	GBS and CIDP: peripheral nerve demyelination with prolonged distal latencies, reduced conduction velocities, conduction block and temporal dispersion. Vasculitic neuropathy: sensory and motor axonal loss localized to individual nerves in a multifocal distribution.
Neuropathology findings	Prominent widespread depletion of myelinated fibres and severe axonal loss.	GBS and CIDP: mononuclear leucocyte infiltration into the endoneurium with macrophage-mediated demyelination. Vasculitic neuropathy: infiltration of peripheral nerve blood vessels by inflammatory cells with endoneurial ischaemia and axonal injury.
Treatment	Symptomatic, neurorehabilitation and podiatrist.	Immunotherapy.

The phenotypic spectrum of *RFC1*-associated disorders continues to expand. Widespread neurodegeneration has been demonstrated with the presence of cerebral and cerebellar atrophy on MRI, increased CSF neurofilament light chain levels and total tau.^8^ Common symptoms include chronic cough, dysarthria, dysphagia, falls, cerebellar and vestibular dysfunction.^2^ Presence of these symptoms may be useful to identify patients who are more likely to have *RFC1*-mediated neuropathy.

Interestingly, some patients with biallelic *RFC1* repeat expansions were reported to respond to immune-therapy.^4^ It is possible that *RFC1*-associated neuropathy can co-exist with immune-mediated processes. The carrier frequency and therefore population prevalence of pathogenic *RFC1* expansions are not insignificant,^9^ and therefore, co-existence with immune-mediated neuropathies should be considered. This has implications for treatment and may be relevant in individuals with known *RFC1* neuropathy who may be progressing faster than expected, or in individuals with diagnosed immune-mediated neuropathy who stop responding to treatment.

The underlying pathophysiology of *RFC1*-associated neuropathy remains unknown. Therefore, it is unclear if carriers of pathogenic *RFC1* expansions could be at risk of neuropathy. Nagy *et al*.^5^ reported that the carrier frequency for pathogenic *RFC1* alleles in their cohort was comparable to controls. This finding contributes to evidence that there is no increased risk of neuropathy in carriers of pathogenic *RFC1* expansions. Further studies into the pathogenesis of *RFC1*-mediated neuropathy will provide insights into the mechanisms of disease and the implications for heterozygous carriers.

In an era of increasing use of genetic testing, identification of patients who are most likely to have a genetic neuropathy is important for efficient utilization of health resources and to minimize side effects of unnecessary investigations and treatments (e.g. sural nerve biopsies, immunotherapy). However, the study by Nagy *et al.* does not support routine testing of the *RFC1* gene in patients with immune-mediated neuropathy. Instead, the best approach may be accurate phenotyping to prioritize those individuals who would benefit from *RFC1* testing.

Overall, Nagy *et al.*’s paper contributes to the growing literature exploring the presence of biallelic pathogenic *RFC1* expansions in various forms of neuropathy. Their findings highlight the importance of clinical assessment and consideration of investigation findings to differentiate *RFC1*-associated neuropathy from immune-mediated neuropathy, a distinction of high clinical relevance given the therapeutic implications.
